# Knowledge, Awareness, and Practices Regarding the Novel Coronavirus Among a Sample of a Pakistani Population: A Cross-Sectional Study

**DOI:** 10.1017/dmp.2020.408

**Published:** 2020-10-23

**Authors:** Saba Tariq, Sundus Tariq, Mukhtiar Baig, Muhammad Saeed

**Affiliations:** 1 Pharmacology, University Medical and Dental College, The University of Faisalabad, Faisalabad, Pakistan; 2 Physiology, University Medical and Dental College, The University of Faisalabad, Faisalabad, Pakistan; 3 Clinical Biochemistry, Faculty of Medicine, Rabigh, King Abdulaziz University, Jeddah, KSA; 4 Clinical Biochemistry, University Medical and Dental College, Faisalabad, Pakistan

**Keywords:** attitude, COVID-19, knowledge, practice, social determinants of health

## Abstract

**Objectives:**

We aimed to investigate knowledge, attitudes, and practices regarding the new coronavirus in a sample of the general Pakistani population.

**Methods:**

This survey was carried out through The University of Faisalabad (TUF), Pakistan, between February 2020 and April 2020. The questionnaire was circulated on various online platforms to gather information. The data were analyzed on SPSS-22.

**Results:**

Out of 2121 respondents (13.7% were male, and 86.3% were females), 7.4% were married, 4.5% had a high income, and 5.8% had fewer family members. Coronavirus disease (COVID-19) knowledge scores were significantly low in the < 21 years age group as compared to the 21 to 25 years age group (*P* < 0.001) and > 25 years age group (*P* < 0.001). The males, married community, high income people, and few family members groups had significantly higher coronavirus knowledge than their respective groups (*P* = 0.033; *P* = 0.001; *P* < 0.001; *P* = 0.042, respectively).

**Conclusion:**

Our findings suggest that the knowledge score among our study population was not up to the mark. However, a positive correlation between the correct knowledge and appropriate attitude and practice was found among study participants. Older age groups and the high income group were associated with adequate knowledge scores.

## Introduction

The novel coronavirus disease (COVID-19) is a highly infectious condition and is declared a global public health emergency by the World Health Organization (WHO).^1^ The first case of COVID-19 was diagnosed in Wuhan City, followed by an outbreak in China.^2^ As of August 16, 2020, there are 21 294 845 confirmed cases throughout the world, and around 187 countries, areas, and territories are affected by this disease.^3^ The case fatality rate of COVID-19 is 4% in China and has been dramatically increased in Italy to 7.7%.^4^ In Pakistan, an early estimation shows that it is spreading rapidly, and up until now, there are 341 070 confirmed cases and 19 492 deaths.^3^

The virus’s ability to reproduce and transmit is estimated through R_0_ value (basic reproductive number), and recent research indicates that coronavirus has an R_0_ average value of around 3.28 with a median of 2.79.^5^ A recent study found an asymptomatic ratio of 41.6% according to screening results obtained from their general population.^6^ As the asymptomatic cases are high, this iceberg of the disease is mainly responsible for the threat to immunocompromised and elderly patients. The fight against COVID-19 has just started and is mainly dependent on individuals’ adherence to preventive and control measures, which are fundamental and are generally influenced by their insight to knowledge, attitudes, and practices (KAP) toward COVID-19.^7^ It is important to understand that disease will remain for a longer period, and people need to learn to start living with the disease and modify their lives accordingly. However, it is vital to break the vicious cycle to avoid exacerbation of the disease.^8^

With the flare-up of coronavirus cases in Pakistan and the whole world, there is an ongoing stage of panic and emotional stress that can positively or negatively affect their knowledge score. Therefore, this study aimed to investigate knowledge, attitude, and different practices of the general population against the COVID-19 pandemic among a sample of the Pakistani population. This study will help the policy-makers and stakeholders rapidly develop a policy to effectively deal with the COVID-19 breakout in a short and supportive way.

## Methods

This questionnaire-based, cross-sectional study was conducted through The University of Faisalabad, Faisalabad. The questionnaire was developed with the help of preliminary research published as a preprint^9^ and was pretested and verified for errors on a group of 50 people from the general population, and modified accordingly. The reliability of the questionnaire was determined by measuring the related Cronbach’s alpha, which was equal to 0.79, indicating good consistency in the responses from study participants. For further improving Cronbach’s alpha, 4 questions were deleted that were affecting its value, and it became equal to 0.82. This study was conducted between February 2020 and April 2020. Due to the increasing number of cases, the lockdown was announced on March 24 in Punjab, Pakistan, resulting in closing all educational institutes and other places where people were gathering. Due to the lockdown, we chose to conduct an online survey to see the knowledge, attitude, and preparedness of the community in the largest populated province of Pakistan Punjab. The sample size was calculated by using OpenEpi calculator.

A consent statement was included at the start of the questionnaire for voluntary participation of the subjects. The questionnaire was circulated on the various platforms (social media and authors’ own network) to gather information. The ethical committee of University Medical and Dental College, TUF, approved the study (TUF/Dean/2020/34). The questionnaire consisted of 4 parts: demographic information, knowledge, attitude, and practice (KAP) questions. The first part has 11 questions regarding the general demographic characteristics and previous history of flu. Regarding knowledge of COVID-19, 10 questions were asked: (1) Is the new coronavirus contagious? (2) Do you think to have a prior health condition, such as heart disease or diabetes, could raise the risk of COVID-19? (3) How would you describe the new coronavirus treatment? (4) Do you think that COVID-19 can be treated at home? (5) Do you think health education can help in the prevention of this disease? (6) Are children also at risk of the new coronavirus infection? (7) Can the novel coronavirus be passed on through food? (8) What should be the safety levels of lab testing for the novel coronavirus? (9) Can a person infected with COVID-19 recover completely and not be infected again? (10) Should the novel coronavirus outbreak concern you about your pets or other animals? For each correct response, 1 mark was awarded, whereas for each incorrect response, zero mark was given. Marks of all 10 responses were summed up for each individual. The attitude was recorded using 6 questions, as given in Table 1, and practice was recorded using 3 questions, as given in Table 2.

**Table 1.** Comparison of COVID-19 knowledge scores among subjects with different levels of perception of various attitudes

Variables	Response	N (%)	Knowledge Score Mean ± SD	*P*-value
What is your perception of the dangerousness of COVID-19?	Seriously dangerous	1337 (63)	5.40 ± 1.58	0.001*
	Very dangerous	402 (19)	5.60 ± 1.49	
	Dangerous	330 (15.6)	5.80 ± 1.62	
	Like the common flu	28 (1.3)	5.46 ± 1.47	
	Not dangerous	24 (1.1)	4.66 ± 1.34	
What is your perception of level of risk of contracting COVID-19 infection?	Most risk	851 (40.1)	5.48 ± 1.57	0.387
	High risk	880 (41.5)	5.53 ± 1.58	
	Moderate risk	269 (12.7)	5.51 ± 1.60	
	Risk similar to that of contracting the common cold	80 (3.8)	5.50 ± 1.56	
	No risk	41 (1.9)	5.02 ± 1.55	
Can eating garlic prevent infection of the new coronavirus?	Yes	313 (14.8)	5.45 ± 1.48	< 0.001*
	No	845 (39.9)	5.87 ± 1.62	
	Not sure	961 (45.3)	5.17 ± 1.49	
Will warm weather stop the outbreak of COVID-19?	Yes	816 (38.5)	5.27 ± 1.48	< 0.001*
	No	669 (31.5)	5.96 ± 1.53	
	Not sure	636 (30.0)	5.27 ± 1.63	
If you knew that there was a 2% chance that animals (eg, camels) are a source for the transmission of the new coronavirus, would you consume camels’ milk or meat?	Yes	130 (6.1)	5.42 ± 1.68	< 0.001*
	No	1626 (76.7)	5.59 ± 1.52	
	Not sure	365 (17.2)	5.07 ± 1.70	
How do you normally treat yourself when you get a cough or fever?	Self-medication like antibiotics	828 (39.0)	5.56 ± 1.58	< 0.001*
	Consult doctor	894 (42.1)	5.31 ± 1.52	
	Google my symptoms	30 (1.4)	4.80 ± 2.00	
	Local remedies like steam	369 (17.4)	5.86 ± 1.55	

**Table 2.** Comparison of COVID-19 knowledge scores among subjects with different practicing behavior in a study population

Variables	Response	N (%)	Knowledge Score Mean ± SD	*P*-value
What is your daily surgical mask use?	Never	345 (16.3)	5.40 ± 1.61	0.425
	Sometimes	1338 (63.1)	5.52 ± 1.59	
	Every day at all times	438 (20.7)	5.47 ± 1.48	
What is your frequency of washing hands per day?	Nil	13 (0.6)	4.46 ± 1.50	< 0.001*
	2-4 times	324 (15.3)	5.21 ± 1.58	
	> 4 times	1784 (84.1)	5.55 ± 1.57	
What is your frequency of using soap for handwashing per day?	Nil	7 (0.3)	4.86 ± 2.03	< 0.001*
	Sometimes	312 (14.7)	5.17 ± 1.67	
	Every time	1802 (85.0)	5.55 ± 1.55	

The data were entered and analyzed in the Statistical Package for Social Sciences (SPSS) version 22 (IBM Corp, Armonk, NY). The normality of the data was checked by Shapiro Wilk statistics. Frequencies and percentages were given for discrete variables, and mean ± SD was given for continuous variables. A comparison between the 2 groups was checked by an independent sample t-test. Comparisons between 3 and more groups were seen using analysis of variance. Individual-level differences or honest significant differences were observed using Tukey’s HSD post hoc analysis. A binary logistic regression analysis of the demographics with adequate knowledge scores was used to find predictors of adequate knowledge scores. For computing binary logistic analysis, the knowledge score was converted into a percentage then into a dichotomous variable by dividing into adequate knowledge (knowledge score > 60%) and inadequate knowledge (knowledge score < 60%).

## Results

The study included 2121 subjects, from the Punjab Province of Pakistan. There were 290 (13.7%) males and 1831 (86.3%) females in this study. The mean age of the study population was 21.8 ± 4.13 years. Subjects falling below 21 years of age were 870 (41.0%), between 21 and 25 years of age were 1089 (51.3%), and above 25 years of age were 162 (7.6%). Table 3 is also showing the comparison of demographic characteristics with COVID-19 knowledge score. The scores were significantly high in males as compared with females (*P* = 0.033). The scores were significantly low in the younger population (*P* < 0.001) and increase as the age increases. A post hoc analysis showed that COVID-19 knowledge scores were significantly low in the < 21 years age group as compared with the 21 to 25 (*P* < 0.001) and > 25 years age group (*P* < 0.001). The scores were significantly high in > 25 years age group (*P* < 0.001) as compared with 21 to 25 (*P* < 0.001) and < 21 years age group.

**Table 3.** General demographic characteristics and their comparison with the COVID-19 knowledge score

Variables		N (%)	Knowledge Score Mean + SD	*P*-value
Gender	Male	290 (13.7)	5.68 ± 1.69	0.033*
	Female	1831 (86.3)	5.46 ± 1.56	
Age (years)	< 21^a^	870 (41.0)	5.19 ± 1.51	< 0.001* a vs b, *P* < 0.001 a vs c, *P* < 0.001 b vs c, *P* < 0.001
	21-25^b^	1089 (51.3)	5.63 ± 1.52	
	> 25^c^	162 (7.6)	6.16 ± 1.90	
Marital status	Married	158 (7.4)	5.91 ± 1.87	0.001*
	Unmarried	1963 (92.6)	5.46 ± 1.54	
Education	Undergraduate^a^	1630 (76.9)	5.46 ± 1.57	0.051 a vs b, *P* = NS^†^ a vs c, *P* = 0.046 b vs c, *P* = NS
	Graduate^b^	349 (16.5)	5.53 ± 1.52	
	Postgraduate^c^	142 (6.7)	5.78 ± 1.69	
Income (PKR/month)	No^a^	1587 (74.8)	5.43 ± 1.53	< 0.001* a vs e, *P* < 0.001 b vs e, *P* = 0.023 d vs e, *P* = 0.021
	< 30 000^b^	134 (6.3)	5.56 ± 1.68	
	30 000 to 50 000^c^	153 (7.2)	5.66 ± 1.75	
	50 001 to 100 000^d^	151 (7.1)	5.57 ± 1.59	
	> 100 000^e^	96 (4.5)	6.19 ± 1.63	
Family members	1-3^a^	124 (5.8)	5.64 ± 1.63	0.042* a vs b, *P* = NS a vs c, *P* = NS b vs c, *P* = NS
	4-6^b^	1367 (64.5)	5.54 ± 1.55	
	> 7^c^	630 (29.7)	5.36 ± 1.61	

^†^NS = nonsignificant.

*Notes*: Superscripts a, b, c, d, e of the items showing a 1-1 comparison of the post hoc test.

The married population has significantly high COVID-19 knowledge scores compared with the unmarried population (*P* < 0.001). The highly educated population also has higher COVID-19 knowledge scores than the less educated population, but the results were non-significant. The population with a high monthly income has higher scores than the population earning less (*P* < 0.001). A post hoc analysis showed that the population whose monthly income was > 100 000 PKR/month has significantly higher scores as compared with the population earning nothing (*P* < 0.001), < 30 000 PKR (*P* = 0.023), and 50 001 to 100 000 PKR/month (*P* = 0.021). The population with fewer family members has significantly high knowledge scores than the population with more family members (*P* = 0.042), but no significance was found on post hoc analysis (see Table 3).

The population who was not sure that they have a family member who had been diagnosed with flu in the years 2019 and 2020 has significantly low knowledge scores (*P* < 0.001 and *P* = 0.006, respectively) as compared with the population who was sure that their family members had or had not developed any kind of flu. Similarly, the population diagnosed with flu in 2020 has higher knowledge scores as compared with the population who was not diagnosed with any type of flu, though this was not significant (*P* = 0.802) (Table 4). The population who was sure that they had close contact with people who had traveled to China or other COVID-19-affected places between November 2019 and March 2020 has significantly high knowledge about COVID-19 compared with the population who was not sure (*P* = 0.022). The population who was not sure about having been immunized with the flu vaccine in the year 2019 has significantly low knowledge about COVID-19 (*P* < 0.001) as compared with the population who was sure (see Table 4).

**Table 4.** Comparison of COVID-19 knowledge scores among subjects with a history of flu

Variables	Response	N (%)	Knowledge Score Mean + SD	*P*-value
Do you have a family member who had been diagnosed with flu in 2019?	Yes	933 (44.0)	5.60 ± 1.55	< 0.001*
	No	847 (39.9)	5.49 ± 1.61	
	Not sure	341 (16.1)	5.20 ± 1.53	
Have you been diagnosed with flu in 2020?	Yes	639 (30.1)	5.51 ± 1.58	0.802
	No	1482 (69.9)	5.49 ± 1.57	
Do you have a family member who had been diagnosed with flu in 2020?	Yes	669 (31.5)	5.52 ± 1.56	0.006*
	No	1209 (57.0)	5.54 ± 1.57	
	Not sure	243 (11.5)	5.19 ± 1.63	
Do you have close contact with people who had traveled to China or other COVID-19-affected places between November 2019 and March 2020?	Yes	160 (7.5)	5.77 ± 1.69	0.018*
	No	1892 (89.2)	5.48 ± 1.56	
	Not sure	69 (3.3)	5.17 ± 1.54	
Have you been immunized with the flu vaccine in 2019?	Yes	208 (9.8)	5.28 ± 1.64	< 0.001*
	No	1720 (81.1)	5.60 ± 1.53	
	Not sure	193 (9.1)	4.79 ± 1.70	

The population who did not consider COVID-19 dangerous has significantly low knowledge about the disease compared with the population who considered the disease dangerous (*P* < 0.001). Regarding the perception of the level of risk of contracting the COVID-19 infection, no significant difference between the knowledge scores was found. The population with the view that eating garlic cannot prevent infection from the new coronavirus has significantly high knowledge scores as compared with the population who said yes (*P* < 0.001) or was not sure (*P* < 0.001). Similarly, the population with the view that warm weather cannot stop the outbreak of the new coronavirus has significantly high knowledge scores as compared with the population who said yes (*P* < 0.001) or was not sure (*P* < 0.001) (see Table 1).

The population with the view of not consuming animal (camel) meat or milk if they knew that there is a 2% chance that animals (camels) are a source for the transmission of the new coronavirus has significantly high COVID-19 knowledge scores as compared with the population who are not sure about this information (*P* < 0.001). The population who self-medicates with antibiotics has significantly high knowledge about COVID-19 compared with the population who Google the symptoms (*P* = 0.045) or consult a doctor (*P* = 0.005). Similarly, the population using local remedies like steam has significantly high knowledge about COVID-19 as compared with the population who Google the symptoms (*P* = 0.002) or consult a doctor (*P* < 0.001), as seen by the post hoc analysis.

The population who never used a surgical mask as a daily practice has low COVID-19 knowledge scores, but the results were not significant (*P* = 0.425). Most of the population 1784 (84.1%) were aware of hand sanitization and were washing hands more than 4 times a day. The COVID-19 knowledge scores were significantly high (*P* < 0.001) in people who washed their hands > 4 times/day. A post hoc analysis showed that people who washed their hands > 4 times/day have significantly high COVID-19 knowledge scores as compared with people who never washed their hands (*P* = 0.034) or washed their hands only 2–4 times/day (*P* = 0.001). Similarly, the population who used soap for handwashing every day has a significantly high knowledge scores as compared with the population who used soap only sometimes for handwashing (*P* < 0.001) (see Table 2).

**Figure 1.**
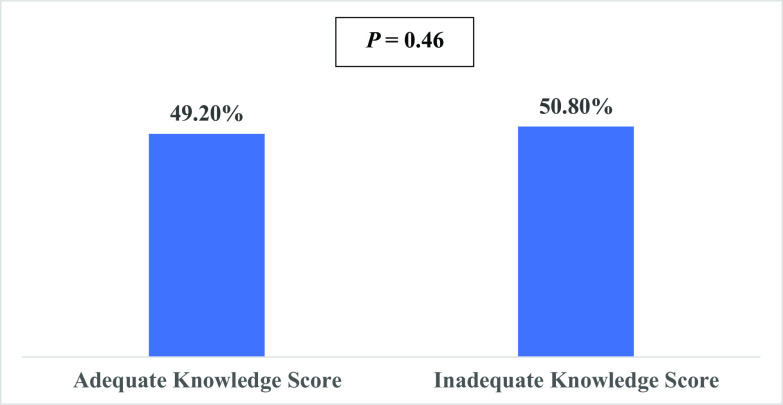
Comparison of knowledge score (≥ 60% adequate knowledge score and < 60% inadequate knowledge score).

We compared the knowledge score (≥ 60% adequate knowledge score and < 60% inadequate knowledge score), but no significant difference was found (*P* = 0.46) (Figure 1). The logistic regression analysis showed that age groups of 21–25 years and > 25 years with OR = 1.49 (*P* < 0.001) and OR = 1.81 (*P* = 0.008), respectively, were more likely to have adequate knowledge about COVID-19 than younger age groups. Similarly, the group with income > 100 000 PKR/month and family members > 6 with OR = 1.61 (*P* = 0.034) and OR = 1.56 (*P* = 0.015), respectively, were more likely to have adequate knowledge toward COVID-19 compared with the lower-income group and fewer family members (Table 5).

**Table 5.** Association of different variables with adequate knowledge score among study participants (binary logistic regression analysis)

	Unstandardized Coefficients		Wald				95.0% Confidence Interval for B	
Variables	B	Std. Error	Beta	df	*P*-value	Exp (B)	Lower Bound	Upper Bound
Female	0.000	0.134	0.000	1	1.000	1.000	0.770	1.299
Married	0.049	0.204	0.058	1	0.810	1.050	0.704	1.567
21-25 years	0.400	0.092	18.734	1	< 0.001	1.491	1.244	1.787
> 25 years	0.596	0.226	6.984	1	0.008	1.815	1.166	2.824
< 30 000 rupees	-0.031	0.185	0.027	1	0.869	0.970	0.676	1.393
30 000 to 50 000 rupees	0.215	0.179	1.447	1	0.229	1.240	0.873	1.760
50 001 to 100 000 rupees	-0.103	0.179	0.332	1	0.564	0.902	0.636	1.280
> 100000 rupees	0.475	0.224	4.482	1	0.034	1.608	1.036	2.496
Family members 4-6	-0.056	0.120	0.220	1	0.639	0.945	0.748	1.195
Family members > 6	0.444	0.182	5.934	1	0.015	1.560	1.091	2.230

## Discussion

This study has highlighted important points regarding KAP among a sample of the Pakistani population. Most of the respondents in our study were young females. We could not find any plausible cause for this gender imbalance. However, one of the interesting findings of our study was that, as the age was increasing, the knowledge score increased significantly, and it was highest in the age group above 25 years. The binary logistic regression analysis also revealed that age groups 21–25 years and > 25 years were significantly associated with the knowledge score. This age-related knowledge score is due to more use of social media in this age group, and this use is further increased due to their confinement at homes because of the lockdown. Although we found more knowledge scores in males, the binary logistic regression analysis revealed no association between knowledge score and gender. In line with our results, another study in China was also conducted to investigate the KAP gap in the general population. In that research, most of the respondents were female, but their female population knowledge score was higher in contrast to our results.^10^ The reason for this difference could be that, in Pakistan, most females remain in houses and do not have social interaction like males; as half of Pakistan’s population are women, it is important to target this population for health education to counter COVID-19 effectively.^11^ Our results also showed that higher education and higher income individuals had significantly more knowledge. Similarly, our regression analysis revealed a direct association of income with knowledge score. One interesting finding was that married people were more knowledgeable as compared with unmarried people. However, we could not find this association in the binary logistic regression analysis. Similar to our results, another study in India demonstrated that higher education and high income were associated with good knowledge. However, surprisingly, concerning practice scores, it was found that lower education and unmarried individuals had good practice regarding COVID-19.^12^

We also found that individuals whose family members were diagnosed with flu or who themselves have had flu had more knowledge. Similarly, those who had contact history with someone coming from China, who considered COVID-19 dangerous, or had a risk of contracting the disease, were more knowledgeable than others. These results highlighted the importance of early dissemination of information and the role of media. The media have been involved in updating information daily regarding the COVID-19 pandemic.^13^ Another study, published as a preprint in Thailand, documented that individuals with a history of flu or any member with flu had more knowledge as they tried to gain more information through different sources.^9^

Regarding attitude, people who were familiar with the myths of eating garlic, warm weather, and food not contaminated with COVID-19 had higher knowledge scores as compared with their counterparts. The reason for this higher knowledge score could be that global giants like America, China, and Europe are already struggling with the disease. Every country, along with the WHO, is giving daily briefings and updates about the disease.^14^ One of the important findings in our post hoc analysis was that individuals who were using surgical masks or washing their hands more than 4 times a day had higher knowledge scores than others. Here again, the role of media and preparedness by the government is very important. In Pakistan, special steps had already been taken by the government to sensitize the individuals regarding COVID-19. In these new measures, media campaigns, posters, banners, local-language messages containing special precautionary reminders are regularly sent on the phone, daily updates on their websites, and mobile apps have been introduced, and the government is playing its role in educating the people.^15^ Although health authorities are taking necessary measures to control the disease, there is a daily increase in cases in most Asian countries, including Pakistan.^16,17^ Due to a shortage of protective equipment like face masks, initially mixed messages were conveyed to the general population by various definitive bodies. However, it was good to observe that people using face masks had more knowledge. In contrast to our results, one of the studies conducted in Malaysia, published as a preprint, showed that people were confused about the use of face masks. It is conceivable that mixed messages prompted the partitioned reaction on the wearing of face masks.^16^

As of now, no vaccine is available for COVID-19. Scientists are working hard to find an effective treatment for this disease. Health catastrophes, such as COVID-19, occur both locally and globally, influencing countries and people’s economic conditions. It seems that awareness of the masses and taking prevention measures are the only hope for controlling and managing this pandemic.^18^ Therefore, a small effort at an individual level to investigate the KAP on COVID-19 among the public is very important to design cost-effective strategies for the long-term fight against the disease. The present study uncovered the requirement for proper dissemination of information. This study will help policy-makers target the population for health education to combat the disease effectively and in the future as well.

## Limitations

Convenient sampling and use of the social media and authors’ network, sampling bias, the dominance of the female gender, and more participants from the younger age group were few limitations of the study. Second, as the study is an online survey, it might not represent the underprivileged people of the community. Those people, unable to afford smartphones and Internet connections, were not included. Our study also excludes the perception of illiterate people.

## Conclusion

Our findings suggest that knowledge score among our study population was not up to the mark. However, a positive correlation between the correct knowledge and appropriate attitude and practice was found among study participants. Older age groups and the group with high income were associated with adequate knowledge scores. Therefore, it is crucial to target a specific part of the community, especially younger age groups, and low-income groups by providing specific health education programs to raise COVID-19 knowledge and improve practices. Such campaigns would play an important role in raising awareness among the targeted groups. We also suggest to include preventive measures against such infectious diseases in the school and college curriculum.
